# Preparation and Nanoencapsulation of Lectin from *Lepidium sativum* on Chitosan-Tripolyphosphate Nanoparticle and Their Cytotoxicity against Hepatocellular Carcinoma Cells (HepG2)

**DOI:** 10.1155/2020/7251346

**Published:** 2020-10-22

**Authors:** Unzila Yasin, Muhammad Bilal, Hamid Bashir, Muhammad Imran Amirzada, Aleena Sumrin, Muhammad Hassham Hassan Bin Asad

**Affiliations:** ^1^Centre for Applied Molecular Biology, University of the Punjab, 87-West Canal, Bank Road Lahore-53700, Pakistan; ^2^Department of Pharmacy, COMSATS University, Islamabad, Abbottabad Campus, 22060, Pakistan; ^3^Institute of Fundamental Medicine and Biology, Department of Genetics, Kazan Federal University, Kazan 420008, Russia

## Abstract

Lectins are the oligomeric sugar-specific glycoprotein of nonimmune origin, are involved in the multiple biological recognition process, and have the capacity to perform a wide variety of physiological functions including antifungal, antiviral, antitumor, and cell agglutination. The main objective of the current study was to prepare lectin protein-loaded chitosan-TPP nanoparticles via ionic gelation methods with different CS/TPP ratios and to investigate anticancer potential against HepG2 cells. The best ratio showed the mean particle size (298.10 ± 1.9 nm, 21.05 ± 0.95 mv) with optimal encapsulation efficiencies of 52.435 ± 0.09%. The cytotoxicity was evaluated against HepG2 cells, and IC_50_ values obtained were 265 *μ*g/ml for lectin protein and 105 *μ*g/ml for lectin-loaded chitosan-TPP nanoparticles, respectively. The mRNA expression of proliferation markers like *GPC3* was significantly decreased in hepatocellular carcinoma cells (HepG2) during lectin protein-loaded chitosan-TPP nanoparticle treatment. Apoptotic genes that indicating a marked increase in expression are *Caspase 3*, *p53*, and *Bax*, while *Bcl2* and *AFP* showed a downregulation of expression after treatment of HepG2 cells with lectin-loaded chitosan-TPP nanoparticles. The preliminary findings of our study highlighted that lectin protein-loaded chitosan-TPP nanoparticles could be a promising anticancer agent.

## 1. Introduction

Hepatocellular carcinoma (HCC), a chronic liver malignancy more prevalent in East with high fatal rate in middle-aged males while being found to be rare in west, has emerged a major health problem [1, 2]. HCC prognosis leads to metastasis aggravating the condition to consider mortality due to significant hepatocarcinogenity. However, the increased survival rate of HCC patients has improved better outcomes with addition of surveillance for the general population [3], improved medication, and advent of newly introduced chemotherapeutic agents like doxorubicin and sorafenib [4]; however, their profounded resistance limits their economical use. Thus, successful antiangiogenesis can be achieved by targeting apoptosis regulating pathways by the antitumor activity of naturally borne biomolecules [5]. Lectins are heterogeneous proteins derived from plants that have been assessed by biochemical characterization to have a strong anticancer effect on HCC [6]. Lectin has been exploited as a cancer biomarker by glycan modification in the preliminary phase of cancer examination [7]. However, lectins' short life limits its commercial use as a cytotoxic molecule. There is a need of identification of novel methods to overcome lectin instability by exploring its anticancerous potential using nanoconstructs [8].

Nanotechnology is found to be widely involved in early cancer detection, diagnosis, and therapy as a secondary alternative to previously reported strategies, i.e., chemotherapy, mediating an effective response only at the initial stages [9, 10]. Chitosan, which is a refined version of chitin, possesses remarkable characteristics of high solubility, efficient delivery at the adsorption site, bioavailability, biocompatibility, and nontoxicity of multiple drug-NP formulations [11]. Of the ligands being employed, polymeric substances are considered the most suitable biomaterial, owing to their biodegradable nature [12]. Metallic ions, mostly anions like dextran and sodium tripolyphosphate (TPP), are gelled with free amino groups of chitosan via electrostatic attraction, formulating different nanoparticle composites by transitional modifications, leading to agglomeration [13, 14]. Colloidal chemistry suggests that chitosan polymer surface has the strong entrapping capacity towards protein and peptides generating a detectable immunogenic response along with enhancing the retention time and activity of the encapsulated drug [15]. Chitosan nanoparticles encapsulated lectin protein (PNPs) inheriting more stability to protein's shelf-life along with rendering high penetrating ability. Chitosan salts are among the most favorable biosurfaces for nanoparticle formulations based on its ability of recognizing and inhibiting metastasized cell proliferation [16]. Future prospect can be accomplished by a combination of different cytotoxic polyanionic molecules with chitosan without altering the covalent integrity of the loaded drug. The present study focuses on the preparation of lectin-loaded chitosan-TPP nanoparticle (PNP) proteins and the exploitation of the cytotoxic potential against hepatocellular carcinoma cell lines (HepG2).

## 2. Materials and Methods

### 2.1. Chemical and Reagents

The polymer chitosan (low molecular weight (% deacetylation 75% to 85%, MW~50 kDa), Cat. No.: 448869) and sodium tripolyphosphate (Cat. No.: 238503) were purchased from Sigma-Aldrich, USA. Q-Sepharose was purchased from Amersham, UK. Ultrapure water was obtained with MilliQ equipment (Waters, USA). All other chemicals and reagents were of analytical grade.

### 2.2. Choice of Fresh Plant

*Lepidium sativum* seeds were collected from the botanical garden of the University of Punjab, Lahore. Seeds were dried at ambient temperature, to avoid moisture infectivity and further to extract the protein.

### 2.3. Isolation of Lectin Protein

*Lepidium sativum* seeds were finely ground and diluted in 50 mM phosphate buffer (pH 7.0) with a ratio of 1 g of powdered seeds in 5 ml buffer and placed at 4°C with continuous stirring for 2 h. The supernatant was passed through a muslin cloth, and homogenate was centrifuged for 15 min at 13000 RPM. The supernatant was saturated with 80% ammonium sulphate with slow stirring at 4°C for 24 h and further centrifuged at high speed for 15 min. Precipitated protein was resuspended in PBS, while total protein concentration was quantified through Bradford and assessed by SDS-PAGE.

### 2.4. Purification of Lectin

Crude protein mixture obtained was initially desalted by employing pD10 desalting column to free protein from salts followed by dialysis using 10 mM phosphate buffer. Purification was carried using anion exchange chromatography by loading the dialyzed protein on Q-Sepharose resin previously equilibrated by 10 mM phosphate buffer (pH 7.4) to remove excessive metabolites.

### 2.5. SDS-PAGE

Mini-protean precast gel (Bio-Rad, Hercules, CA) was used in SDS-PAGE. The rest of all procedure was the same as described by Asad et al. [17]. Eluted protein fractions were run on 12% SDS-PAGE.

### 2.6. Bradford Method for Protein Estimation

Shortly, 150 *μ*l of reagents at 37°C was mixed with sample for 120 min in a 96-well plate. Later on, at 562 nm, absorbance was measured via Tecan infinite M200 pro plate reader (Magellan 7) at ambient temperature. Protein strength in different samples was estimated via a standard curve for BSA [18]. Fractions showing positive hemagglutination assay were pooled together, and concentration was estimated via this method.

### 2.7. Hemagglutinating Activity Assay

In order to evaluate the erythroagglutination effect of lectin protein, a 96-well microtiter plate was used. Twofold serial dilution of *Lepidium sativum* (50 *μ*l) purified lectin protein was mixed with 2% human erythrocyte solution (50 *μ*l) prepared with PBS in 96-well plates and incubated for 2 h at 37°C. The results were evaluated to determine the minimum concentration exhibiting hemagglutination activity. Hemagglutination titer, the reciprocal of the maximum dilution showing hemagglutination is considered to be a single hemagglutination unit [19].

### 2.8. Lectin Protein-Loaded Chitosan-TPP Nanoparticle Preparation

In order to prepare the chitosan nanoparticles through an ionic gelation method, TPP was used as a cross-linking agent [20]. The 0.5% (*w*/*v*) storage solution of chitosan was prepared to dissolve the chitosan in DDW containing 1.2% acetic acid and by adjusting to pH 5.4 using NaOH, while TPP stock solution of 0.5% (*w*/*v*) concentration in DDW was prepared and filtered through a 0.25 *μ*m sterile filter. Preparing the lectin-loaded protein chitosan-TPP nanoparticle by the ionic gelation method and optimizing the concentration ratio of chitosan and TPP for improved particle size and entrapment efficiency, 1 mg of lyophilized lectin protein was added to 1 ml of chitosan nanoparticles with varying concentrations (0.2, 0.3, and 0.4% (*w*/*v*)) and mixed, while TPP solution with concentrations was added to the chitosan lectin protein mixed solutions using a continuous stirring syringe (800 rpm) with a magnetic stirrer at room temperature (30°C). All dilutions were stirred for 20 min at 800 rpm followed by centrifugation at high speed to collect the particles. The lectin protein-loaded chitosan-TPP nanoparticles were dissolved in distilled water and used for further analysis.

### 2.9. Entrapment Efficiency of Chitosan Nanoparticles

Bradford assay was used to evaluate the chitosan-TPP nanoparticle entrapment efficiency of target protein by measuring the absorption at 595 nm of free lectin protein obtained in a transparent supernatant after nanoparticle separation. Entrapment efficiency was calculated as follows:

(1) $$\begin{array}{c} \text{Entrapment efficiency}:\frac{\text{Total protein used in formulation}-\text{free amount of protein}}{\text{Total protein used in the formulation}}\times 100. \end{array}$$

### 2.10. Evaluation of Size and Zeta Potential of Lectin-Loaded Nanoparticles

The detailed characterization of nanoparticles (control) and lectin-loaded nanoparticles was performed by zeta sizers (from Comsats University, Abbottabad) to determine its mean size (nm), zeta potential (mV), and polydispersity index using dynamic light scattering (DLS) at a detection angle of 90° at 25°C. In order to calculate the zeta potential diluted nanoparticles, the solution was evaluated in triplicate by putting it in a universal folded capillary cuvette equipped with a platinum electrode for measurement and results were reported as the mean ± SD.

### 2.11. In Vitro Lectin Release

The release of lectin in PBS (pH 7.4) was investigated from the lectin nanoparticle complex. The lectin nanoparticle complex was suspended in PBS (pH 7.4) and incubated at 37C. The sample was drawn at regular intervals of time such as 6, 12, 24, 48, and 96 h, followed by centrifugation at 13000 rpm for 25 min. Released lectin concentration was found by Bradford assay [18].

### 2.12. Cell Culture and Cytotoxicity Analysis

Human hepatocellular carcinoma cell lines (HepG2) were provided by ATCC and preserved in CAMB, University of Punjab, and Lahore, Pakistan. Dulbecco's modified Eagle medium was used for cell culturing additionally supplemented with 10% fetal bovine serum (FBS), antibiotics (penicillin, streptomycin), and essential amino acid incubated in a cell culture incubator under a humified atmosphere.

### 2.13. Evaluation of HepG2 Cell Viability by MTT Assay

HepG2 cell line was used to evaluate the cytotoxicity of lectin-loaded chitosan-TPP nanoparticles by MTT assay [21]. A color difference between yellow and purple was found to estimate increased cell viability of lectin protein activity [22]. Cells were cultured in DMEM media in 96-well plates under humified conditions of 37°C for 72 h. Different concentrations of lectin protein (19 to 450 *μ*g/ml), twofold serial dilutions of chitosan-TPP nanoparticles (100 to 6.3 *μ*g/ml), and lectin-loaded chitosan-TPP nanoparticles (150 to 4.7 *μ*g/ml) were plated in triplicate along with control against HepG2 cells and reincubated for 24 h at 37°C. The media was changed after 24 h, followed by an addition of 10 *μ*l of MTT reagent (5 mg/ml), incubated for 4 h. Subsequently, a 100 *μ*l DMSO reagent was added to dissolve purple insoluble MTT-formazan crystals at the base of the wells to form a single cell suspension, and absorbance was measured at 570 and 630 nm [23].

### 2.14. Expression Study of Cancer Biomarkers by Lectin-Loaded Chitosan-TPP Nanoparticles

Expression studies of anticancer activity of lectin-loaded chitosan nanoparticles were conducted by examining mRNA expressions of *Bax*, *Bcl2*, *AFP*, *p53*, *GPC3*, and *Caspase 3* genes in HepG2-treated cells. To conduct the study, HepG2 cells were cultured in 6-well plates for 24 hours with the known concentration (20 & 40 *μ*g/ml) of lectin-loaded chitosan-TPP nanoparticles using 24-well microtiter plates. After incubation, cells were subjected to isolation of total RNA and cDNA synthesis, thus examining the expression of above mentioned genes using the SYBR™ Green master mix (Thermo Fisher, Lithuania) as fluorescent dye and *β* actin as the reference gene by quantitative RT-PCR.

### 2.15. Real-Time PCR for Gene Expression Analysis

Using an RNA extraction kit (Qiagen, Germany), total RNA was extracted from lectin-loaded nanoparticle-treated HepG2 cells. Extracted RNA was estimated by Nanodrop, and cDNA of isolated RNA was synthesized using cDNA synthesis kit (Thermo Fisher, USA) as instructed by the manufacturer. Expression analyses were evaluated of the selected genes involved in HCC, and qPCR was carried out using a synthesized cDNA template with the primers listed in Table 1. Relative quantification of selected genes was performed by means of real-time PCR and *β actin* as the reference gene.

### 2.16. Statistical Analysis

Statistical analyses were carried out with GraphPad Prism 7. An experiment was conducted in triplicate, and descriptive statistics was used to determine the mean and standard deviation of the data. One-way ANOVA was used to explore the significant reduction in mRNA expression of the different genes (*p* ≤ 0.05).

## 3. Results

### 3.1. Isolation and Purification of Lectin Protein

**Figure 1:**
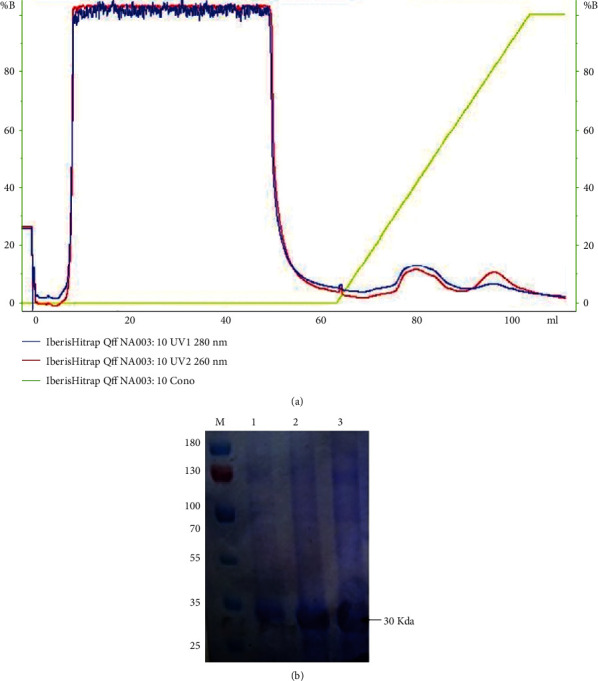
SDS-PAGE of the purified fractions of lectin protein from Lepidium sativum seed by ion exchange chromatography

Lectin protein was purified from the seeds of *Lepidium sativum* with 80% ammonium sulphate saturation followed by anion exchange chromatography. Crude protein from seeds were extracted in sodium phosphate buffer (pH 7.0) and dialyzed in 10 mM phosphate buffer followed by purification on Q-Sepharose resin. Purified fractions of the 30 kDa band (analyzed on SDS-PAGE) with lectin activity were pooled (Figure 1).

### 3.2. Hemagglutination Assay

The presence of active lectin in purified protein was monitored by hemagglutination inhibition assay. Trypsin-treated blood was tested to detect an affinity of lectin protein towards human erythrocytes. A hemagglutinin test was carried in a 96-well plate with human RBCs; RBC surface bearing sugars act as a probe for lectin carbohydrate recognition domain, accounting validity of oligosaccharides towards lectin protein. Lectin protein reveals hemagglutinating activity against human erythrocytes at 20 *μ*g/ml. *Lepidium sativum* lectin retained full hemagglutinating activity at pH 7∼8 and temperature 30∼40°C. The formation of the carpet layer at the bottom of wells indicates the inhibition of agglutination by lectin-oligo specificity, while nonagglutinated RBCs were clumped, leading to a red dot, indicating a lack of lectin protein.

### 3.3. Entrapment Efficiency

The lectin purified from the seeds of *Lepidium sativum* was successfully entrapped by ionic gelation methods on the chitosan-TPP nanoparticle matrix. Lectin protein encapsulation efficiency in the nanoparticle was directly related to the cross-linking agent in the solution. Results obtained indicate low entrapment efficiency about 43.891 ± 0.63 in case of 0.2/0.04 chitosan/TPP ratio, while 0.4/0.095 chitosan/TPP ratio resulted in high encapsulation, i.e., 56.54% ± 1.4 for lectin protein.

### 3.4. Zeta Size and Potential of Lectin Protein-Loaded Chitosan Nanoparticle

**Figure 2:**
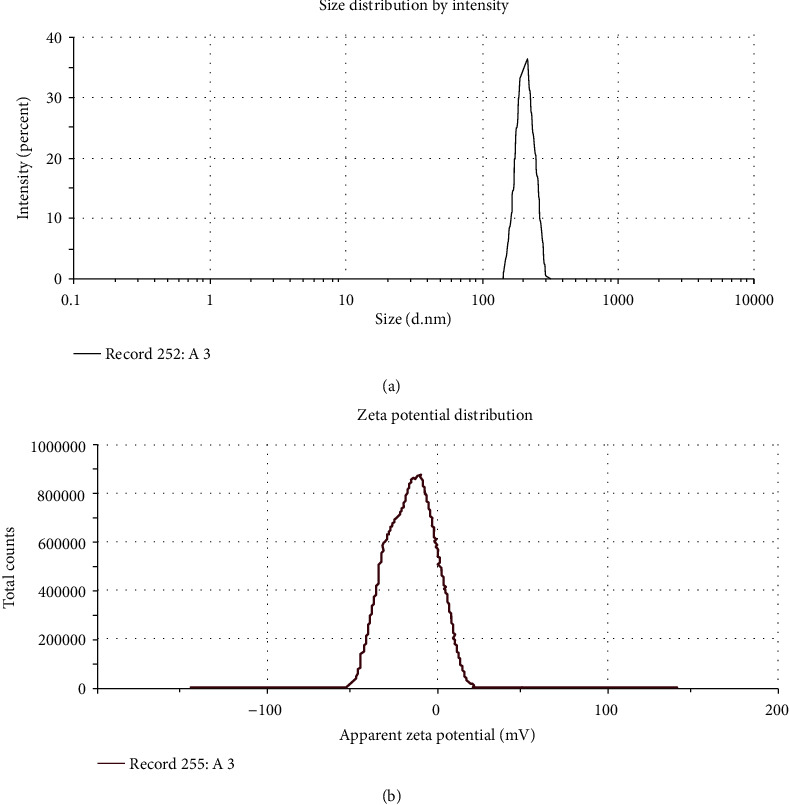
Characteristics of lectin protein-loaded chitosan-TPP nanoparticles (a, b).

Particle size, surface charge, and morphology play a fundamental role in the cellular uptake of nanoparticle. Zeta potential plays an imperative role in efficacy, targeting, and stability in nanoparticle formulations [24]. Lectin protein-loaded chitosan-TPP nanoparticles were formulated by using a certain amount of chitosan (containing 1 mg of the lectin protein) and TPP. To optimize the size and entrapment efficiency, it was observed that with the increase in TPP volume or the decrease in the chitosan/TPP ratio resulted in an increase of the size of particle while the zeta potential decreases relative to the increase in TPP concentration. From the data, we concluded that the increase in chitosan to TPP ratio directly influences the formation of larger particles and increases the efficiency of entrapment. The concentration of TPP has an influential effect on the nanoparticle polydispersity. The size and entrapment efficiency of nanoparticles prepared from the different formulations are shown in Table 2 and Figure 2.

### 3.5. In Vitro Lectin Release

**Figure 3:**
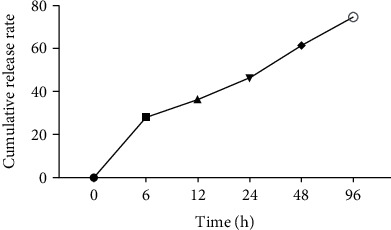
Lectin protein released profile from the nanoparticle lectin complex (SD was 0.005 for each concentration).

The cumulative release of lectin (%) ranged from 12.3 to 74.8% as presented in Figure 3. Lectin concentrations such as 28.4, 36.2, 46.5, 61.7, and 74.8% were measured at 6, 12, 24, 48, and 96 h, respectively. Our findings show the preliminary accelerated burst release of lectin from the nanoparticle lectin complex. At some later stages, the release declined to a slower rate for 24 h and a total release of 61.7% in 48 h and 74.8% in 96 h.

### 3.6. Cytotoxicity Potential of Lectin-Loaded Chitosan-TPP Nanoparticles

**Figure 4:**
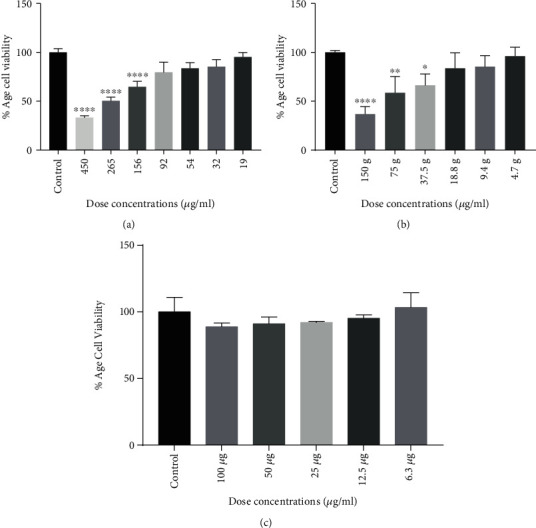
Cytotoxic effect of lectin (a), lectin protein-loaded chitosan-TPP nanoparticles (b), and chitosan-TPP nanoparticles (c) on HepG2 cells. Bars indicate standard deviation. ∗, ∗∗, ∗∗∗, and ∗∗∗∗ indicate the significance level at *p* < 0.5, *p* < 0.01, *p* < 0.001, and *p* < 0.0001.

The cytotoxicity of chitosan-TPP nanoparticles loaded with lectin protein was assessed against HepG2 cells. The viable cell count depends upon its mitochondrial count indicating total cell population. In living cells, mitochondrial succinic dehydrogenase enzyme catalyzes the conversion of yellow MTT compound to purple MTT-formazan crystals. However, this quantitative cytotoxicity assay signifies the formation of soluble formazan product accounted by a multiwell scanning spectrophotometer, showing cell viability efficiency [25] (Figure 4). After 24 h, cell viability was evaluated using different concentrations of lectin protein (19-450 *μ*g/ml). Control used was without lectin protein-loaded chitosan-TPP nanoparticle-treated HepG2 cells with 100% cell viability. One-way ANOVA shows the significant cytotoxic effect at 156, 265, and 450 *μ*g/ml at *p* < 0.0001 with cell viability inhibition of 36%, 50%, and 67%, respectively, as compared to the control. The calculated value for IC_50_ was 265 *μ*g/ml. Chitosan-TPP nanoparticles showed the no significant cytotoxic effect at maximum concentration used up to 100 *μ*g/ml, while lectin protein-loaded chitosan-TPP nanoparticles showed the significant cytotoxic effect at 37.5 (*p* < 0.5), 75 (*p* < 0.01), and 150 *μ*g/ml (*p* < 0.0001) with cell viability inhibition 34%, 42%, and 64%, respectively, as compared to the control, and IC_50_ was calculated as 105 *μ*g/ml.

### 3.7. Gene Expression in Lectin Protein-Loaded Chitosan-TPP Nanoparticle-Treated Cells

**Figure 5:**
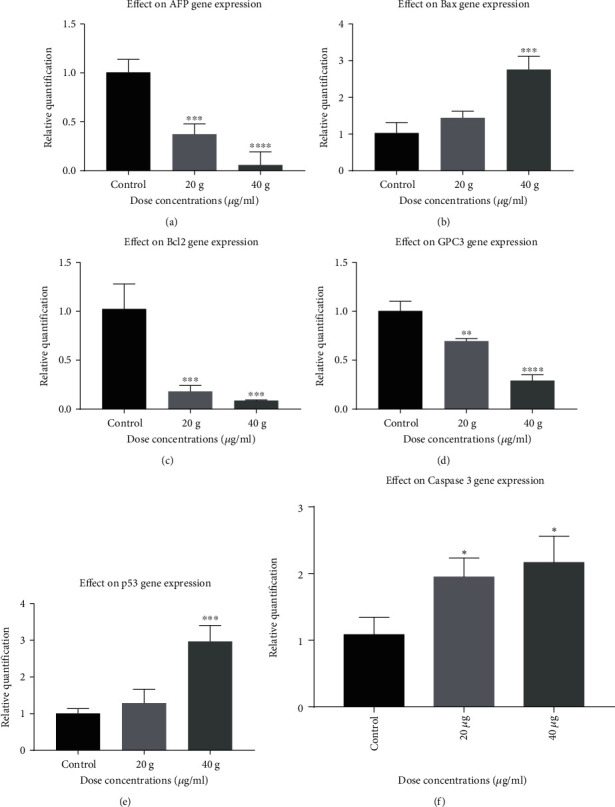
Expression analysis of the selected genes with respect to the control. Results of gene expression analyses via RT-qPCR showed that the lectin-loaded chitosan-TPP nanoparticles caused statistically significant upregulated expression of *Bax*, *Caspase 3*, and p53, while genes showed downregulated expression of *Bcl2*, *AFP*, and *GPC-3*.

A gene expression study highlights the apoptotic mode of action of lectin-loaded chitosan-TPP nanoparticles towards genetic biomarkers tested along beta actin as the housekeeping gene, ensuring the arrest of proliferation and metastasis in response to its treatment. Lectin protein-loaded chitosan-TPP nanoparticle-treated HepG2 cells indicate a significant upregulated expression of *Bax* (*p* < 0.001), *Caspase 3* (*p* < 0.01), and *p53* (*p* < 0.001) whereas contrary to it, *Bcl2* (*p* < 0.0001), *GPC-3* (*p* < 0.001), and *AFP* (*p* < 0.0001) showed downregulated expression after lectin-loaded chitosan-TPP nanoparticle treatment. Results indicated a direct inhibitory action of lectin-loaded chitosan-TPP nanoparticles on the targeted HepG2 cell lines. Expressional variation of *AFP*, *p53*, *Bax*, *Caspase 3*, *GPC-3*, and *Bcl2* genes upon lectin-loaded chitosan-TPP nanoparticle treatment is shown in Figure 5.

## 4. Discussion

Lectins are plant defense proteins that mediate immune responses through their carbohydrate recognition domains. These proteins have specialty to bind certain specific sugars, glycoprotein, and glycolipids, displaying a variety of physiological attributes like antifungal [26], antiviral [27], anticancerous [28, 29], cell agglutination [30], and immune modulatory effects [31]. In general, lectins isolated from plants have been extensively used in immunology and cell biology for diagnostic, therapeutic purpose as well as an immune regulatory agent [32, 33]. Extensive researches stated that few lectins have been isolated rendering promising antitumor activities such as a lectin Con A (concanavalin A) from Jack bean and ML-I lectin from mistletoe, currently practiced in preclinical trials for the management of malignant melanoma and human liver cancer [29, 34]. However, the use of the protein and peptides as a therapeutic agent is restricted due their chemical and physical instabilities by the enzymatic degradation or some other environmental changes [35]. In recent decades, researchers have studied the difference in the patterns of cytotoxicity of lectin proteins once encapsulated in various core materials [36]. Statements endorsing the chitosan nanoparticles to entrap lectin exploit its potential to improve protein stability against adverse conditions [37]. Lectins are investigated in combination with nanoparticles, in order to exploit their diagnostic and therapeutic use at the clinical level in the fields of oncology, immunology, and cell biology [38].

The main goal of the proposed study was to isolate the lectin protein from *Lepidium sativum* seeds and to synthesize the lectin-loaded chitosan-TPP nanoparticles. The isolated lectin protein and the synthesis lectin-loaded chitosan-TPP nanoparticles were used to evaluate their anticancer potential against HepG2cells. Several authors have suggested various plant lectin isolation techniques based on classical purification methods. In this study, we purified 30 kDa lectin protein using an anion exchange chromatography from *Lepidium sativum* seeds as previously purified through affinity chromatography and confirmed by a hemagglutination assay [39]. A similar 32 kDa lectin protein has been reported from Curcuma seeds purified by gel filtration and affinity chromatography [40]. As a result, several strategies employed for lectin protein isolation and purification from a variety of plant sources have been identified. Multiple approaches have been put in place for drug delivery systems to resolve the issues regarding the stability of anticancer proteins. In the present study, we used the ionic gelation method for lectin protein loading on chitosan by the use of anionic cross-linker (TPP) in acidic conditions. The chitosan/TPP nanoparticle cross-linking structure is mainly pH dependent and corresponds to the reaction between TPP ions and NH3+ group of chitosan [41]. Bhattarai et al. reported that changing physical conditions and the parameters like cross-linker/polymer and drug ratio influences the entrapment efficiency [42]. In the nanoencapsulation of lectin protein in chitosan-TPP nanoparticles by increasing the chitosan/TPP ratio 0.2/0.04 to 0.4/0.095, the entrapment efficiency of the lection protein was obtained ranging from 43.891 ± 0.63 to 52.435 ± 0.09%. These findings might be due to the high level of interacting units due to the increase of polymer/TPP ratio resulting in an increase of the size of the particle and entrapment efficiency [43, 44]. Similar results of loaded nanoparticles with the BSA and estradiol have been reported [45, 46]. The successful formulation of nanoparticles relies on its ability for entrapping. Nanoparticle entrapment efficiency was found to increase with increasing the chitosan to TPP ratio as observed in the formulation of chitosan hydrogel beads [44, 47] and polyethylene glycol-coated gold nanoparticles [48]. Of course, many plant lectins are anticancer, and their loading on chitosan-NPs makes them more potent. By encapsulating nanoparticles, this characteristic of lectins protein is further improved. Chitosan nanoparticles were assessed for their therapeutic potential against A549 cancer cell lines. The lowest cell viability was observed with exposure to 200 *μ*g/ml chitosan nanoparticles, with a gradient decrease in cell viability [49]. We aimed to determine the cell toxicity of lectin protein, chitosan nanoparticles, and lectin protein-loaded chitosan-TPP nanoparticles against HepG2 cell lines. The significant cytotoxic effect of lection protein was observed at 156, 265, and 450 *μ*g/ml at *p* < 0.0001. The calculated value of lectin protein for IC_50_ was 265 *μ*g/ml. Chitosan-TPP nanoparticles showed no significant cytotoxic effect at maximum concentration used (100 U/ml), while lectin protein-loaded chitosan-TPP nanoparticles showed the significant cytotoxic effect at 37.5 (*p* < 0.5), 75 (*p* < 0.01), and 150 *μ*g/ml (*p* < 0.0001), and IC_50_ was calculated as 105 *μ*g/ml. With various clinical trials, this is widely accepted for now that plant lectins are involved in apoptosis induction by modifying cell signaling pathways involving *Bcl-2* family, caspase family, *p13K/Akt*, *p53*, *Ras-Raf*, *BNIP3*, *ERK*, and ATG families in cancer [50, 51]. A diverse set of apoptosis-specific genes, including *p53*, *Bax*, *AFP*, and *Caspase 3*, was considered candidates for an expression study along with a nonapoptotic gene *Bcl-2*. The tendency of lectin protein-loaded chitosan-TPP nanoparticles to regulate mRNA proliferation varies with differently expressed stable genes. Significant upregulation in *p53*, *Bax*, and *Caspase 3* was observed in lectin protein-loaded chitosan-TPP nanoparticle-treated HepG2 cells, whereas an antagonistic effect was noticed in *AFP* and *Bcl-2*, indicating a decrease expression in a dose-dependent manner.

## 5. Conclusions

Lectin-loaded chitosan-TPP nanoparticles with a different CS/TPP ratio having anticancer potential against HepG2 cells were investigated. Cytotoxicity (IC_50_) against HepG2 cells (265 *μ*g/ml) for lectin and lectin-loaded chitosan-TPP nanoparticles (105 *μ*g/ml) were recorded significant. Apoptotic genes that were marked increased in expression included *Caspase 3*, *p53*, and *Bax*, while *Bcl2* and *AFP* showed a down regulation of expression after treatment of HepG2 cells with lectin-loaded chitosan-TPP nanoparticles. It is therefore concluded that lectin-loaded chitosan-TPP nanoparticles could be a promising anticancer agent in the future.

## Figures and Tables

**Table 1:** List of primers used for expression studies.

Genes	Primers	Sequence
*AFP*	Forward	5′-TGTCCCTCCTGCATTCTCTG-3′
	Reverse	5′-TGGCAGCATTTCTCCAACAG-3′
*GPC3*	Forward	5′-TACTGCTCTTACTGCCAGGG-3′
	Reverse	5′-ACCAAGCAGTACGTTCTCCA-3′
*Bax*	Forward	5′-GGAGGATTGTGGCCTTCTTT-3′
	Reverse	5′-GCCGTACAGTTCCACAAAGG-3′
*Caspase 3*	Forward	5′-GAGGCCGACTTCTTGTATGC-3′
	Reverse	5′-AATTCTGTTGCCACCTTTCG-3′
*Bcl-2*	Forward	5′-ACCAAGAAGCTGAGCGAGTC-3′
	Reverse	5′-AAGTAGAAAAGGGCGACAACC-3′
*Beta actin*	Forward	5′-AGAGCTACGAGCTGCCTGAC-3′
	Reverse	5′-AGCACTGTGTTGGCGTAGAC-3′
*p53*	Forward	5′-CCAGTGTGATGATGGTGAGG-3′
	Reverse	5′-ACCAAGAAGCTGAGCGAGTC-3′

**Table 2:** Characteristics of lectin protein-loaded chitosan nanoparticle entrapment efficiency, size, zeta potential (+mv), and polydispersion index (PDI < 0.45) analysis of nanoparticles (mean ± SD).

Chitosan (%, *w*/*v*)	TPP (*μ*l)	TPP (%, *w*/*v*)	Chitosan (%, *w*/*v*)/TPP (%, *w*/*v*)	Entrapment efficiency (%)	Size (nm)	Zeta potential (±mv)	PDI
0.2%	0.1	0.04	0.2/0.04	43.891 ± 0.63	143.525 ± 1.09	36.99 ± 1.09	0.306
	0.12	0.05	0.2/0.05	49.896 ± 0.24	171.0 ± 2.7	26.45 ± 0.85	0.371
0.3%	0.15	0.06	0.3/0.06	45.639 ± 0.31	178.98 ± 5.9	27.51 ± 0.50	0.315
	0.18	0.07	0.3/0.07	52.435 ± 0.09	216.105 ± 4.0	22.83 ± 0.86	0.425
0.4%	0.20	0.085	0.4/0.085	48.118 ± 1.09	225.91 ± 2.1	26.23 ± 0.68	0.370
	0.24	0.095	0.4/0.095	56.54 ± 1.4	298.10 ± 1.9	21.05 ± 0.95	0.468

## Data Availability

Data was generated at the Center for Applied Molecular Biology, University of the Punjab, Lahore, Pakistan, and could be requested from the shared corresponding author Dr. Hamid Bashir.
